# Genetic variants in *FBLIM1* gene do not contribute to SAPHO syndrome and chronic recurrent multifocal osteomyelitis in typical patient groups

**DOI:** 10.1186/s12881-020-01037-7

**Published:** 2020-05-12

**Authors:** Gunter Assmann, Michaela Köhm, Volker Schuster, Frank Behrens, Rotraut Mössner, Nina Magnolo, Vinzenz Oji, Harald Burkhardt, Ulrike Hüffmeier

**Affiliations:** 1 Department of Internal Medicine I, José-Carreras Centrum for Immuno- and Gene Therapy, University of Saarland Medical School, /Saar, Homburg, Germany; 2 Division of Rheumatology and IME, Fraunhofer Institute, Branch for Translational Medicine and Pharmacology, Goethe University, Frankfurt am Main, Germany; 3 Hospital for Children and Adolescents, University of Leipzig, Leipzig, Germany; 4 Department of Dermatology, Georg-August-University Göttingen, Göttingen, Germany; 5 Department of Dermatology, University of Münster, Münster, Germany; 6 Institute of Human Genetics, University Hospital Erlangen, Friedrich-Alexander-Universität Erlangen-Nürnberg, Schwabachanlage 10, 91054 Erlangen, Germany

**Keywords:** Chronic recurrent multifocal osteomyelitis (CRMO), Chronic non-bacterial osteomyelitis (CNO), Syndrome of synovitis acne pustulosis hyperostosis osteitis (SAPHO (syndrome)), Coding variants, Association

## Abstract

**Background:**

Syndrome of synovitis acne pustulosis hyperostosis osteitis (SAPHO) and chronic recurrent multifocal osteomyelitis (CRMO) present two diseases of a dermatologic and rheumatologic spectrum that are variable in manifestation und therapeutic response. Genetic risk factors have long been assumed in both diseases, but no single reliable factor has been identified yet. Therefore, we aimed to clinically characterize a patient group with syndrome of synovitis acne pustulosis hyperostosis osteitis (SAPHO) (*n* = 47) and chronic recurrent multifocal osteomyelitis (CRMO)/ chronic non-bacterial osteomyelitis (CNO) (*n* = 9) and analyze a CRMO candidate gene.

**Methods:**

Clinical data of all patients were collected and assessed for different combinations of clinical symptoms. SAPHO patients were grouped into categories according to the acronym; disease-contribution by pathogens was evaluated. We sequenced coding exons of *FBLIM1*.

**Results:**

Palmoplantar pustular psoriasis (PPP) was the most common skin manifestation in CRMO/CNO and SAPHO patients; most SAPHO patients had sterno-costo-clavicular hyperostosis. The most common clinical category of the acronym was S_PHO (*n* = 26). Lack of pathogen detection from bone biopsies was more common than microbial isolation. We did not identify autosomal-recessive *FBLIM1* variants.

**Conclusions:**

S_PHO is the most common combination of symptoms of its acronym. Genetic analyses of *FBLIM1* did not provide evidence that this gene is relevant in our patient group. Our study indicates the need to elucidate SAPHO’s and CRMO/CNO’s pathogenesis.

## Background

S*yndrome ofsynovitisacnepustulosishyperostosisosteitis* (SAPHO) belongs to a group of rare, variable, chronic autoinflammatory diseases of the skeleton often in combination with certain skin manifestations and typically manifests in early adulthood [1–3]. SAPHO syndrome’s manifestations of the skeleton are manifold; however, osteitis with hyperostosis is considered as a pathognomonic lesion of SAPHO syndrome [3]. Moreover, it can present as a sclerosing osteitis originating from the bone marrow, a hyperostosis with increased bone cuff formed by the periost, an ossification of ligaments, accompanied by osteolysis or an erosion of a joint. Predilection sites of the disease are joints/ bones of the anterior chest wall, mostly sterno-costo-clavicular structures. As skeletal manifestations in SAPHO syndrome often affect the spine and have common features with ankylosing spondylitis and psoriatic arthritis (PsA), it has been considered to belong to the spondylarthropathies [3].

*Chronicrecurrentmultifocalosteomyelitis* (CRMO) has a similar clinical spectrum, and the typical range of manifestation in CRMO is childhood [4–6]. CRMO lesions affect most frequently the lower extremity, the vertebrae, pelvic girdle and the foot [6]. Chronic non-bacterial osteomyelitis (CNO) is the more comprehensive name for this entity, so rarely used, while comprising milder/ unifocal osteomyelitis forms also [7].

Palmoplantar pustular psoriasis (PPP) is the most common skin manifestation in CRMO and SAPHO syndrome [1, 8]. Although the further typical skin manifestation of acne contributed to the name SAPHO syndrome, it is less frequently observed, at least simultaneously, while often prior to skeletal manifestations. SAPHO syndrome patients can also manifest with the more common psoriasis form, psoriasis vulgaris (PsV). Similarly as in CRMO, the frequency of Crohn’s disease is increased in SAPHO [1, 8, 9] and considerably higher than one might expect when combining the prevalences of the two diseases. The significant clinical overlap between CRMO and SAPHO causes recurrent discussions whether CRMO represents the pediatric manifestation of SAPHO; and a transition from CRMO to adult SAPHO syndrome has been described in several cases [5, 10–12].

The etiology of CRMO and SAPHO syndrome is unsolved. Current hypotheses in SAPHO suggest a genetic predisposition in combination with a bacterial infection, resulting in reactive osteitis [3, 13]. Interestingly, the pathogen *Propionibacterium acnes* could be isolated in patients of several independent studies, e.g. [1, 8, 9, 14]. In previous genetic studies of SAPHO, single candidate genes – partially known to be causal in clinically overlapping, but syndromic forms (e.g. Majeed syndrome) or in mice models of the diseases - were analyzed in single patients/ families with SAPHO or SAPHO similar symptoms, but disease-causing mutations were not identified [15, 16].

More recently, bi-allelic rare variants of *FBLIM1* encoding filamin binding LIM protein 1 were identified in two CRMO patients: one homozygous missense variant was selected as the most plausible candidate of homozygous variants in 22 genes [17]. One of further 96 CRMO patients was a compound-heterozygous carrier of a heterozygous frameshift variant/ an intronic variant located (rs41310367) in a putative enhancer. An intronic *FBLIM1* variant was observed to be more frequent in CRMO patients than in the general population (rs114077715). Findings of this new potential candidate gene *FBLIM1* in CRMO prompted us to analyze our cohort of CRMO and SAPHO syndrome patients for rare coding variants in *FBLIM1*.

## Methods

All patients were of European origin and diagnosed with CRMO/ CNO or SAPHO syndrome at German university hospitals by board certified rheumatologists, pediatricians, specialized in pediatric rheumatology (*n* = 51), or by dermatologists (*n* = 5). Data on clinical characteristics were collected. For the assignment of SAPHO syndrome patients to the different combinations of its acronym, we omitted two patients due to lack of some essential data. Study approval was obtained through the ethical committees of the Universities of Erlangen, Frankfurt, Homburg and Göttingen; all individuals provided their written informed consent and in the case of minors, written informed consent was obtained from their legal guardians. All investigations were conducted according to the Declaration of Helsinki principles.

Coding exons and adjacent intronic sequences of *FBLIM1* were sequenced by Sanger using intron based primers (Supplementary Table 1, Supplementary Fig. 1, Supplementary Table 3) as described recently [18]. Published candidate variants of a frequency of > 2% prompted us to consider all variants with a minor allele frequency (MAF) of < 3%; we compared their frequency with the frequency of the largest publicly available group of European controls (gnomAD) [19] (https://gnomad.broadinstitute.org/) using allele frequency distribution tests.

## Results

### Clinical characteristics of patient groups

**Fig. 1:**
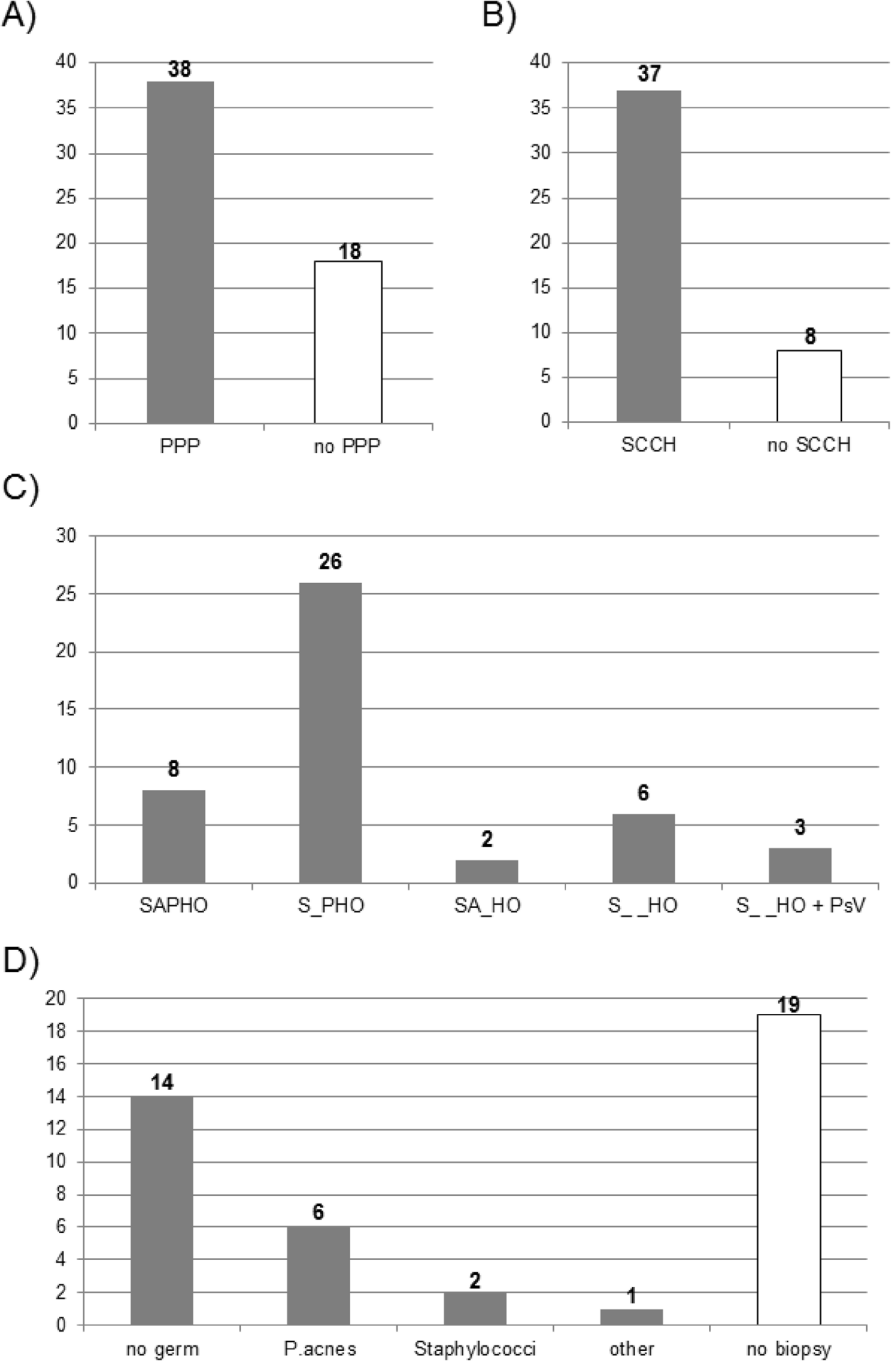
Clinical characteristics in CRMO and SAPHO syndrome patients. **a** Number of patients with/ without palmoplantar pustular psoriasis (PPP). **b** Number of SAPHO syndrome patients with sterno-costo-clavicular hyperostosis (SCCH). **c** Number of SAPHO syndrome patients fulfilling all or part of the clinical characteristics included in the acronym SAPHO (syndrome of synovitis acne pustulosis hyperostosis osteitis). **d** Number of bone biopsies performed in 23 SAPHO syndrome patients with (lack of) detection of germs and number of patients (*n* = 19) who did not have bone biopsies. P. acnes = *Proprionibacterium acnes.*

The majority of 9 CRMO/ CNO and 47 SAPHO syndrome patients were female (*n* = 35; 62%). The average age of onset in CRMO/ CNO patients was 12.2 (± 4.6) years, in SAPHO syndrome 40.2 (± 14.1) years (Supplementary Table 2A). The majority of all patients had PPP (*n* = 38; 68%; Fig. 1a), 24% acne.

Eight CRMO/ CNO patients had multifocal osteomyelitis; and frequencies of skin manifestations are described in Supplementary Table 2B. Two CRMO patients had additional Crohn’s disease, manifesting only 1–2 years later than CRMO.

In 47 SAPHO syndrome patients, the majority had sterno-costo-clavicular hyperostosis (SCCH) (*n* = 37; 82%, Fig. 1b; Supplementary Table 2A). When considering typical manifestations of SAPHO designating its acronym (syndrome of synovitis acne pustulosis hyperostosis osteitis), 58% of 45 patients (*n* = 26; Fig. 1c) matched the category of S_PHO, 18% (*n* = 8) the full acronym and 4% (*n* = 2) SA_HO. 13% of patients (*n* = 6) did neither have PPP nor acne, while in 7% of patients (*n* = 3), plaque type psoriasis as a single manifestation was diagnosed. PsV was more commonly observed in patients also presenting with PPP (*n* = 8) than as a single skin manifestation (*n* = 3). Arthritis was commonly diagnosed in 69% of SAPHO syndrome patients. In SAPHO syndrome patients, 74% (25 of 34) of patients with PPP had additional arthritis, while arthritis without PPP was observed in 55% (6 of 11). While data on family history was not available for many patients, most SAPHO syndrome patients had a negative family history. In 26% of patients with detectable germ in bone biopsy (*n* = 23), *P. acnes* was identified, while germs were not discovered in 61% of biopsied patients (Fig. 1d).

### Analysis of variants in the *FBLIM1* gene

We sequenced *FBLIM1* with a genotyping rate of 100% (Supplementary Table 3). We did not identify any rare (< 0.1%) or truncating variant. Three variants had a MAF of > 3% (Table 1) with a similar allele frequency in patients compared to control individuals [19]. Four further variants had a MAF of 1.85–2.65% in the control individuals. Two variants were located in introns, at positions of − 32 (rs41310367) and − 29 (rs144567113). A further variant was synonymous (c.447G > A/ p.Ala149Ala; rs140170023) at a phylogenetically not conserved position. Last but not least, we identified rs114077715, a variant that is non-synonymous in a single (NM_001024215) of several isoforms of *FBLIM1*. Carriers of RARE variants were exclusively SAPHO syndrome patients.

**Table 1:** Identified variants in coding sequences and adjacent introns of *FBLIM1*, their allele frequencies in the largest group of European control individuals (gnomAD) and allele counts and frequencies in the group of CRMO and SAPHO syndrome patients as derived by i.a. individual patient data presented in Supplementary Table 3.

Position chromo-some 1 (hg19)	16,091,760	16,095,031	16,096,893	16,096,934	16,101,217	16,101,332	16,111,014
**dbSNP-ID (fre-quency of >/< 3%)**	rs41310367 (rarer)#	rs140170023 (rarer)	rs12146078 (frequent)	rs10927851 (frequent)	rs41268337 (frequent)	rs114077715 (rarer)#	rs144567113 (rarer)
**Position in gene/ functional effect**	intronic	coding/ synonymous	Intronic	coding/ missense	coding/ synonymous	intronic or coding/ missense	intronic
	c.250 + 32C > T (NM_001024216, NM_001024215, NM_017556)	c.251-1873G > A (NM_001024216) or c.447G > A/ p.Ala149Ala (NM_001024215, NM_017556)	c.251-11C > T (NM_001024216) or c.542-11C > T (NM_001024215, NM_017556)	c.281C > T/p. Ser94Phe (NM_001024216) or c.572C > T/ Ser191Phe (NM_001024215, NM_017556)	c.525C>T/ p.Cys175Cys (NM_001024216) or c.816C>T/p.Cys272Cys (NM_001024215, NM_017556)	c.599 + 41G > A (NM_001024216) or c.890 + 41G > A (NM_017556) or c.931G > A/ p.Gly311Arg (NM_001024215)	c.718-29C > T (NM_001024216), c.1009-29C > T (NM_017556)
**No. of NFE alleles in gnomad**	116,018	62,210	66,198	66,614	66,672	125,834	66,454
	C	G	C	C	C	G	C
Allele frequencies	97.36%	98.15%	78.87%	31.05%	89.75%	97.35%	97.89%
	T	A	T	T	T	A	T
	2.64%	1.85%	21.13%	68.95%	10.25%	2.65%	2.11%
**56 patients with CRMO/SAPHO**	C	G	C	C	C	G	C
	109 (97.32%)	108 (96.43%)	87 (77.68%)	33 (29.46%)	103 (91.96%)	111 (99.11%)	110 (98.21%)
Allele counts and frequencies	T	A	T	T	T	A	T
	3 (2.68%)	4 (3.57%)	25 (22.32%)	79 (70.54%)	9 (8.04%)	1 (0.89%)	2 (1.79%)

# variants considered as contributing variants by Cox et al. [17]; *NFE* group of non-Finnish European individuals [19].

All but one carrier of the rarer variants carried a single variant, while one patient carried the two variants rs41310367 and rs140170023 in heterozygous state, respectively. Although we cannot exclude a functional role of the second variant, a synonymous variant is not an obvious functional candidate. The overall frequency of all identified variants was comparable to a large group of European control individuals (62,210–125,834 Non-Finnish European alleles; Table 1) [19].

## Discussion

The clinical picture in CRMO and SAPHO syndrome has previously been described as variable, while certain features show comparable frequencies [1, 4–6, 8, 20]. Concordantly, most of our patients had ≥1 additional skin manifestation. The finding of the most common combination “S_PHO” might be related to our predominant recruitment by rheumatologists. Recently, we observed that by recruiting PPP patients by dermatologists, 25% of patients had additional arthritis [21]. When applying previous established diagnostic criteria for SAPHO [2, 3], these PPP patients could be diagnosed to have SAPHO and correspond to a subgroup of patients with synovitis and PPP (S_P_ _). Interestingly, we obtained evidence that several manifestations (PPP, PsV, arthritis) in SAPHO syndrome manifest more often in combination than as single symptoms. Overall, the distribution of clinical symptoms in our study is comparable to previous patient groups and therefore representative for these diseases.

In contrast to the previous study on *FBLIM1*, we did not identify any rare missense or truncating variants or any evidence for carriers of two decent candidate variants. Previously, rs114077715 or a genetic variant in linkage disequilibrium (LD) was suggested to be a potential disease-contributing variant in carriers [17], while the lower frequency of this variant in patients compared to controls in our analyses does not confirm the variant’s or an LD-dependent variant’s relevance. Cox et al. [17] provided evidence that the rare allele of the other variant rs41310367 reduces binding to a transcription factor using in vitro experiments. Our data does not exclude the minor allele of rs41310367 as a potential disease-contributing variant in carriers, but our findings do not implicate a major role of this variant and indicate lack of other candidate variants in coding/ near-coding regions of the gene in this independent patient group. The South-Asian origin of the two patients carrying more critical variants [17] might suggest a relevance of this gene in patients of Asian, rather than of European origin, while frequencies of the rare SNPs identified in this study are comparable in South-Asian probands in gnomAD [19].

Cox et al. [17] considered *FBLIM1* as a suitable candidate gene for CRMO due to a murine knockout model [22]. In those mice, loss of the protein Fblim1 impaired growth and survival of bone marrow stromal cells in vitro, increased osteoclast differentiation in vivo and the level of receptor activator of nuclear factor κB ligand (RANKL), suggesting that Fblim1 might be a major regulator of bone homeostasis. Current findings in human patients with SAPHO syndrome and CRMO – combining the previous study and our one - indicate that only a minority of patients carries genetic variants in the corresponding human gene that are functional.

We cannot exclude that lack of confirmation of *FBLIM1* as a relevant gene might be due to our smaller patient group or smaller proportion of CRMO patients. Still, when considering SAPHO syndrome and CRMO as part of the same disease spectrum, our study does not support *FBLIM1* as a disease gene. As the pathogenesis of CRMO and SAPHO syndrome is not well understood, further genetic and immunologic studies are needed to elucidate their molecular basis. This will be fundamental for therapeutic strategies.

## Conclusions

Our study indicates that the combination of symptoms representing S_PHO instead of SAPHO is the most common subcategory. Autosomal-recessive variants in the *FBLIM1* gene did not play a role in our typical patients.

## Supplementary information

**Additional file 1.**

## Data Availability

The datasets generated and/or analyzed during the current study are available in this manuscript and its supplementary information files, see also Supplementary Table 3 and Supplementary Fig. 1. We used data of the publicly available database “Genome Aggregation Database (gnomAD)” to assess the frequency of the largest publicly available group of Europeans [19] (https://gnomad.broadinstitute.org/). Datasets used and/ or analyzed during the current study are available from the corresponding author on reasonable request.

## Abbreviations

CRMO: Chronic recurrent multifocal osteomyelitis
gnomAD: Genome Aggregation Database (gnomad.broadinstitute.org)
FBLIM1: Filamin binding LIM protein 1
MAF: Minor allele frequency
P. acnes: *Proprionibacterium acnes*
PPP: Palmoplantar pustular psoriasis
PsA: Psoriatic arthritis
PsV: Psoriasis vulgaris
SAPHO syndrome: Syndrome of synovitis acne pustulosis hyperostosis osteitis
SCCH: Sterno-costo-clavicular hyperostosis
