# Supplementary material for: Genetic variants in FBLIM1 gene do not contribute to SAPHO syndrome and chronic recurrent multifocal osteomyelitis in typical patient groups
Source: BMC Med Genet. 2020 May 12;21:102. doi: 10.1186/s12881-020-01037-7 (PMC7216525; doi:10.1186/s12881-020-01037-7)

**Supplementary data to:**

**Genetic variants in *FBLIM1* gene do not contribute to SAPHO syndrome and chronic recurrent multifocal osteomyelitis in typical patient groups**

**Supplementary Table 1: Primer sequences used to sequence *FBLIM1***

| **Primer name** | **Primer sequence** |
| --- | --- |
| FBLIM1-Ex01F | GTCGTCCAGGGAACAAAGG |
| FBLIM1-Ex01R | CAAGAGACTGGGACGCAAAG |
| FBLIM1-Ex02F | CCGGAGCCTAGCCCTGA |
| FBLIM1-Ex02R | ACAGGGGTGAGACCCAAAA |
| FBLIM1-Ex03F | CCTGGTGGGTCCTGTCTTC |
| FBLIM1-Ex03R | TGTGTGAATTTTGTCAGGATGA |
| FBLIM1-Ex04F | CAGCAAATCAGTGGCAGAGT |
| FBLIM1-Ex04R | GCAGAAGAATGCACCAGACA |
| FBLIM1-Ex05F | ACCACCCAGATGAGGAACAG |
| FBLIM1-Ex05R | GATGGGAAGAGGGGTCCT |
| FBLIM1-Ex06F | CTCGGGTATCCTGAGCATGT |
| FBLIM1-Ex06R | CTCATCTGTGAAAGGGGTCA |
| FBLIM1-Ex07F | GGCAGCCTCTCAGAAGAAGA |
| FBLIM1-Ex07R | ATGGGGTTTTACCGTATTGG |
| FBLIM1-Ex07SeqF* | CTTGAGTCCTGGGTGCTGG |
| FBLIM1-Ex08F | CAGACATCTCTGCAGTCCCATA |
| FBLIM1-Ex08R | GTCCAAGCAACCCAGCTAA |
| FBLIM1-Ex09F | CACTTAGGAAGGGCATCCAC |
| FBLIM1-Ex09R | GGAAACCAAGTCAGGGAAGG |

* Primer FBLIM1-Ex07SeqF was used to sequence the PCR product in F direction.

**Supplementary Table 2: Detailed clinical characteristics of A) 47 SAPHO syndrome patients and B) 9 CRMO/ CNO patients**

**A)**

| **Feature** | **No of individuals (percentage)** |
| --- | --- |
| Gender – male / female | 18 (38) / 29 (62) |
| Average of onset (years) | 40.2 ± 14.1 |
| SCCH – yes / no | 37 (82 ) / 8 (18) # |
| Palmoplantar pustular psoriasis – yes / no | 35 (74) / 12 (26) |
| Acne – yes / no | 11 (24) / 35 (76) + |
| Psoriasis vulgaris – yes / no | 11 (24) / 34 (76) # |
| CRMO – yes / no | 5 (12) / 38 (88) “ |
| Arthritis – yes / no | 33 (70) / 14 (30) |
| Family history – positive / negative | 5 (20) / 20 (80) & |

# 2x unknown, + 1x unknown, “ 4x unknown, & 22x unknown

**B)**

| **Feature** | **No of individuals (percentage)** |
| --- | --- |
| Gender – male / female | 3 (33) / 6 (67) |
| Average of onset (years) | 12.2 ± 4.6 |
| CRMO/ CNO – unifocal / multifocal | 1 (11) / 8 (89) |
| Palmoplantar pustular psoriasis – yes / no | 3 (33) / 6 (67) |
| Acne – yes / no | 2 (22) / 7 (78) |
| Psoriasis vulgaris – yes / no | 4 (44) / 5 (56) |
| Crohn’s disease – yes / no | 2 (22) / 7 (78) |
| Family history – positive / negative | 3 (50) / 3 (50) # |

# 3x unknown

**Supplementary Table 3: Detailed genetic and clinical data of the 56 patients.**

f= female, m= male, mf= multifocal, n=no, na= not applicable, neg.= negative, P. acnes = *Proprionibacterium acnes,* pos.= positive, PPP= palmoplantar pustular psoriasis, SCCH= sterno-costo-clavicular hyperostosis, thorac.= thoracic, u= unknown, uf= unifocal, y=yes.

|  | **rs41310367** | [**rs140170023**](https://www.ncbi.nlm.nih.gov/SNP/snp_ref.cgi?type=rs&rs=rs140170023) | [**rs12146078**](https://www.ncbi.nlm.nih.gov/SNP/snp_ref.cgi?type=rs&rs=rs12146078) | [**rs10927851**](https://www.ncbi.nlm.nih.gov/SNP/snp_ref.cgi?type=rs&rs=rs10927851) | [**rs41268337**](http://www.ncbi.nlm.nih.gov/projects/SNP/snp_ref.cgi?rs=rs41268337) | **rs114077715** | [**rs144567113**](https://www.ncbi.nlm.nih.gov/SNP/snp_ref.cgi?type=rs&rs=rs144567113) | **gender** | **age of onset (in years)** | **PPP** | **Psoriasis vulgaris** | **Acne** | **CRMO** | **Osteitis** | **Arthritis** | **Crohn's disease** | | **family history** | **bone biopsy, detection of germ** |
| --- | --- | --- | --- | --- | --- | --- | --- | --- | --- | --- | --- | --- | --- | --- | --- | --- | --- | --- | --- |
| SAPHO-01 | C/C | G/G | C/T | T/T | C/C | G/G | C/C | m | 19 | n | n | n | n | SCCH | y | na | neg. | | no biopsy |
| SAPHO-02 | C/C | G/G | C/T | T/T | C/C | G/G | C/C | f | 73 | y | n | n | n | left 4th rip | n | na | neg. | | no biopsy |
| SAPHO-03 | C/C | G/G | C/C | C/C | C/T | G/G | C/C | m | 34 | n | n | n | n | SCCH | y | na | pos. | | no biopsy |
| SAPHO-04 | C/C | G/G | C/T | C/T | C/C | G/G | C/C | f | 54 | n | y | n | n | thorac. spine | n | na | neg. | | no biopsy |
| SAPHO-05 | C/C | G/G | C/C | T/T | C/C | G/G | C/C | m | 38 | y | y | y | n | SCCH bilateral | y | na | neg. | | no biopsy |
| SAPHO-06 | C/C | G/G | C/C | T/T | C/C | G/G | C/C | m | 34 | n | y | y | n | SCCH | y | na | neg. | | no germ |
| SAPHO-07 | C/C | G/G | C/T | T/T | C/C | G/G | C/C | f | 50 | n | y | n | y | SCCH | y | na | pos. | | no biopsy |
| SAPHO-08 | C/C | G/G | C/C | C/C | C/C | G/G | C/C | f | 41 | y | n | y | n | SCCH | y | na | neg. | | no germ |
| SAPHO-09 | C/C | G/A | C/T | C/T | C/C | G/G | C/C | m | 23 | n | n | y | n | SCCH right | n | na | neg. | | no biopsy |
| SAPHO-10 | C/C | G/G | T/T | T/T | C/C | G/G | C/C | f | 43 | y | y | n | n | SCCH | y | na | neg. | | no biopsy |
|  | **rs41310367** | [**rs140170023**](https://www.ncbi.nlm.nih.gov/SNP/snp_ref.cgi?type=rs&rs=rs140170023) | [**rs12146078**](https://www.ncbi.nlm.nih.gov/SNP/snp_ref.cgi?type=rs&rs=rs12146078) | [**rs10927851**](https://www.ncbi.nlm.nih.gov/SNP/snp_ref.cgi?type=rs&rs=rs10927851) | [**rs41268337**](http://www.ncbi.nlm.nih.gov/projects/SNP/snp_ref.cgi?rs=rs41268337) | **rs114077715** | [**rs144567113**](https://www.ncbi.nlm.nih.gov/SNP/snp_ref.cgi?type=rs&rs=rs144567113) | **gender** | **age of onset (in years)** | **PPP** | **Psoriasis vulgaris** | **Acne** | **CRMO** | **Osteitis** | **Arthritis** | **Crohn's disease** | **family history** | | **bone biopsy, detection of germ** |
| SAPHO-11 | C/C | G/G | C/C | C/T | C/C | G/G | C/C | f | 49 | y | y | n | n | SCCH bilateral | y | na | neg. | | Staphylococci |
| SAPHO-12 | C/T | G/A | C/C | C/C | C/C | G/G | C/C | f | 26 | y | n | y | n | SCCH | y | na | neg. | | no biopsy |
| SAPHO-13 | C/C | G/G | C/C | C/T | C/T | G/G | C/C | m | 45 | y | n | y | n | SCCH bilateral | y | na | neg. | | no biopsy |
| SAPHO-14 | C/C | G/G | C/C | C/T | C/C | G/G | C/C | f | 26 | y | n | y | n | SCCH | y | na | neg. | | no biopsy |
| SAPHO-15 | C/C | G/G | C/T | T/T | C/C | G/G | C/C | f | 59 | y | n | n | n | SCCH | y | na | neg. | | no biopsy |
| SAPHO-16 | C/C | G/G | C/C | C/T | C/C | G/G | C/C | f | 19 | y | y | y | y | SCCH bilateral, mandibula | y | na | neg. | | no germ |
| SAPHO-17 | C/C | G/G | C/C | C/T | C/C | G/G | C/C | m | 57 | y | n | n | n | SCCH bilateral | y | na | u | | no biopsy |
| SAPHO-18 | C/C | G/G | C/C | C/T | C/C | G/G | C/C | m | 7 | n | n | n | y | os ileum | y | na | neg. | | no germ |
| SAPHO-19 | C/C | G/G | C/C | C/T | C/T | G/G | C/C | m | 35 | n | n | n | n | os ileum | y | na | neg. | | no biopsy |
| SAPHO-20 | C/C | G/G | C/T | T/T | C/C | G/G | C/C | f | 33 | y | y | n | n | SCCH bilateral | n | na | neg. | | no biopsy |
| SAPHO-21 | C/C | G/G | C/C | C/T | C/C | G/G | C/C | f | 54 | y | n | n | n | SCCH bilateral | y | na | u | | no biopsy |
| SAPHO-22 | C/C | G/G | C/T | C/T | C/T | G/G | C/C | f | 38 | y | y | y | n | SCCH | y | na | pos. | | no biopsy |
| SAPHO-23 | C/C | G/G | C/T | C/T | C/T | G/G | C/C | f | 41 | y | n | n | n | SCCH left | y | na | neg. | | no germ |
|  | **rs41310367** | [**rs140170023**](https://www.ncbi.nlm.nih.gov/SNP/snp_ref.cgi?type=rs&rs=rs140170023) | [**rs12146078**](https://www.ncbi.nlm.nih.gov/SNP/snp_ref.cgi?type=rs&rs=rs12146078) | [**rs10927851**](https://www.ncbi.nlm.nih.gov/SNP/snp_ref.cgi?type=rs&rs=rs10927851) | [**rs41268337**](http://www.ncbi.nlm.nih.gov/projects/SNP/snp_ref.cgi?rs=rs41268337) | **rs114077715** | [**rs144567113**](https://www.ncbi.nlm.nih.gov/SNP/snp_ref.cgi?type=rs&rs=rs144567113) | **gender** | **age of onset (in years)** | **PPP** | **Psoriasis vulgaris** | **Acne** | **CRMO** | **Osteitis** | **Arthritis** | **Crohn's disease** | **family history** | | **bone biopsy, detection of germ** |
| SAPHO-24 | C/C | G/G | C/T | T/T | C/C | G/G | C/C | f | 17 | y | n | n | n | SCCH | y | na | u | | no germ |
| SAPHO-25 | C/C | G/G | C/C | T/T | C/C | G/G | C/C | m | 28 | y | n | n | n | SCCH | n | na | u | | P.acnes |
| SAPHO-26 | C/C | G/G | C/C | T/T | C/C | G/G | C/C | f | 52 | y | n | n | n | SCCH | y | na | u | | P.acnes |
| SAPHO-27 | C/C | G/G | C/T | T/T | C/C | G/G | C/C | m | 39 | y | n | n | n | SCCH bilateral | n | na | u | | no biopsy |
| SAPHO-28 | C/C | G/G | C/C | C/T | C/C | G/G | C/C | m | 64 | y | n | n | n | SCCH, spine | n | na | u | | P.acnes |
| SAPHO-29 | C/C | G/G | C/T | T/T | C/C | G/G | C/C | m | u | y | n | n | n | SCCH, sternum | y | na | u | | P.acnes |
| SAPHO-30 | C/C | G/G | C/T | T/T | C/C | G/G | C/C | m | 28 | y | n | n | n | SCCH | y | na | u | | P.acnes |
| SAPHO-31 | C/C | G/G | C/C | C/C | C/T | G/G | C/C | f | 42 | n | n | n | n | sternum | n | na | u | | no germ |
| SAPHO-32 | C/C | G/G | C/T | T/T | C/C | G/G | C/T | f | 35 | n | n | n | n | os ileum | n | na | u | | no germ |
| SAPHO-33 | C/C | G/A | C/C | C/T | C/C | G/G | C/C | f | 60 | n | n | n | n | SCCH | n | na | u | | no germ |
| SAPHO-34 | C/C | G/G | T/T | T/T | C/C | G/G | C/C | m | 59 | y | n | n | n | SCCH | y | na | u | | Staphylococci |
| SAPHO-35 | C/C | G/G | C/T | T/T | C/C | G/G | C/C | f | 44 | y | n | n | n | sternum | n | na | u | | other |
|  | **rs41310367** | [**rs140170023**](https://www.ncbi.nlm.nih.gov/SNP/snp_ref.cgi?type=rs&rs=rs140170023) | [**rs12146078**](https://www.ncbi.nlm.nih.gov/SNP/snp_ref.cgi?type=rs&rs=rs12146078) | [**rs10927851**](https://www.ncbi.nlm.nih.gov/SNP/snp_ref.cgi?type=rs&rs=rs10927851) | [**rs41268337**](http://www.ncbi.nlm.nih.gov/projects/SNP/snp_ref.cgi?rs=rs41268337) | **rs114077715** | [**rs144567113**](https://www.ncbi.nlm.nih.gov/SNP/snp_ref.cgi?type=rs&rs=rs144567113) | **gender** | **age of onset (in years)** | **PPP** | **Psoriasis vulgaris** | **Acne** | **CRMO** | **Osteitis** | **Arthritis** | **Crohn's disease** | **family history** | | **bone biopsy, detection of germ** |
| SAPHO-36 | C/C | G/G | C/C | T/T | C/C | G/G | C/C | f | 51 | y | n | n | n | spine | n | na | u | | P.acnes |
| SAPHO-37 | C/C | G/G | C/C | C/T | C/T | G/G | C/C | f | 21 | y | n | n | n | SCCH | y | na | u | | no germ |
| SAPHO-38 | C/T | G/G | C/C | C/C | C/C | G/G | C/C | f | 31 | y | n | n | n | os ileum | n | na | u | | no germ |
| SAPHO-39 | C/C | G/G | C/T | T/T | C/C | G/G | C/C | f | 48 | y | n | n | n | SCCH, sternum | y | na | u | | no germ |
| SAPHO-40 | C/C | G/A | C/C | C/C | C/C | G/G | C/C | m | 53 | y | n | n | n | spine | n | na | u | | no germ |
| SAPHO-41 | C/T | G/G | C/C | C/C | C/C | G/G | C/C | f | 49 | y | n | n | y | spine | y | na | u | | no germ |
| SAPHO-42 | C/C | G/G | C/C | T/T | C/C | G/G | C/C | m | 46 | y | y | n | y | SCCH, left femur | y | na | neg. | | u |
| SAPHO-43 | C/C | G/G | C/C | T/T | C/C | G/A | C/C | f | 38 | y | u | y | u | SCCH | y | na | neg. | | u |
| SAPHO-44 | C/C | G/G | C/C | T/T | C/C | G/G | C/C | m | 40 | y | u | u | u | SCCH | y | na | pos. | | u |
| SAPHO-45 | C/C | G/G | C/C | T/T | C/C | G/G | C/C | f | 44 | y | n | n | n | SCCH, left 1st rip, 4th/5th lumbar spinal body | y | na | pos. | | no biopsy |
| SAPHO-46 | C/C | G/G | C/T | T/T | C/C | G/G | C/C | f | u | n | n | n | u | u | y | na | u | | u |
| SAPHO-47 | C/C | G/G | C/C | T/T | C/C | G/G | C/C | f | 21 | y | y | y | u | u | y | na | u | | u |
| CRMO-01 | C/C | G/G | C/C | T/T | C/T | G/G | C/T | f | 18 | y | y | n | uf | mandibula | y | u | u | | u |
|  | **rs41310367** | [**rs140170023**](https://www.ncbi.nlm.nih.gov/SNP/snp_ref.cgi?type=rs&rs=rs140170023) | [**rs12146078**](https://www.ncbi.nlm.nih.gov/SNP/snp_ref.cgi?type=rs&rs=rs12146078) | [**rs10927851**](https://www.ncbi.nlm.nih.gov/SNP/snp_ref.cgi?type=rs&rs=rs10927851) | [**rs41268337**](http://www.ncbi.nlm.nih.gov/projects/SNP/snp_ref.cgi?rs=rs41268337) | **rs114077715** | [**rs144567113**](https://www.ncbi.nlm.nih.gov/SNP/snp_ref.cgi?type=rs&rs=rs144567113) | **gender** | **age of onset (in years)** | **PPP** | **Psoriasis vulgaris** | **Acne** | **CRMO** | **Osteitis** | **Arthritis** | **Crohn's disease** | **family history** | | **bone biopsy, detection of germ** |
| CRMO-02 | C/C | G/G | C/T | T/T | C/C | G/G | C/C | f | 16 | y | y | n | mf | na | na | n | pos. | | u |
| CRMO-03 | C/C | G/G | C/C | T/T | C/C | G/G | C/C | f | 17 | y | n | n | mf | na | na | u | u | | u |
| CRMO-04 | C/C | G/G | C/C | C/T | C/T | G/G | C/C | m | 10 | n | n | n | mf | na | na | u | u | | u |
| CRMO-05 | C/C | G/G | C/T | C/T | C/C | G/G | C/C | m | 13 | n | n | y | mf | na | na | u | pos. | | u |
| CRMO-06 | C/C | G/G | C/T | T/T | C/C | G/G | C/C | f | 6 | n | n | n | mf | na | n | n | neg. | | u |
| CRMO-07 | C/C | G/G | C/C | C/T | C/C | G/G | C/C | f | 11 | n | y | y | mf | na | n | y | neg. | | u |
| CRMO-08 | C/C | G/G | C/T | C/T | C/C | G/G | C/C | m | 14 | n | n | n | mf | na | n | y | neg. | | u |
| CRMO-09 | C/C | G/G | C/C | T/T | C/C | G/G | C/C | f | 5 | n | y | n | mf | na | n | n | pos. | | u |

**Supplementary Figure 1: Sanger-sequences of seven SNPs described in Table 1 in all patients shown as (A) six representative electropherograms and (B) as a general overview of sequence alignments.** Arrows indicate the SNP in (A); dot/ cross below the sequence alignments and the marked nucleotide (black/ brown/ yellow) represent the SNP in (B).


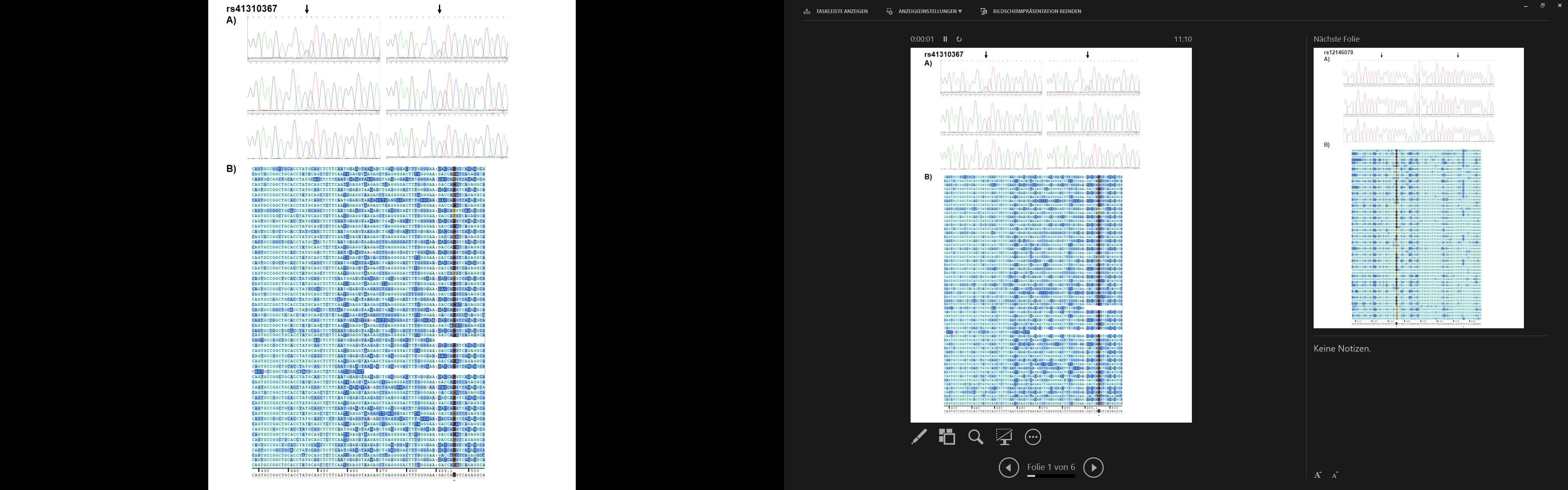


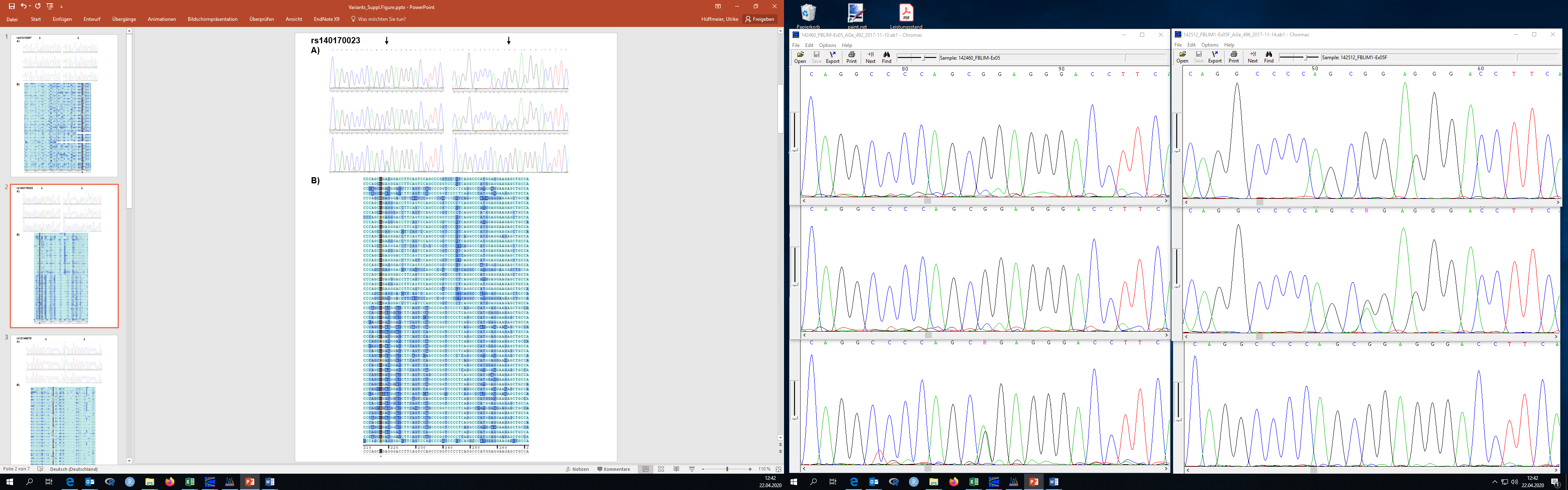


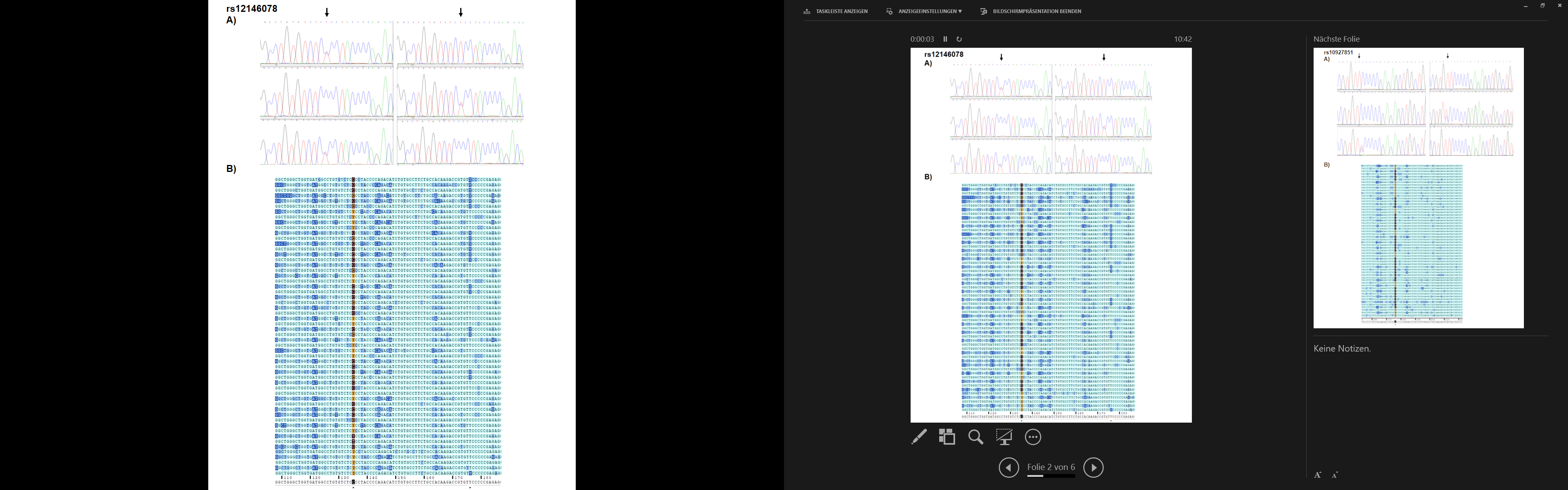


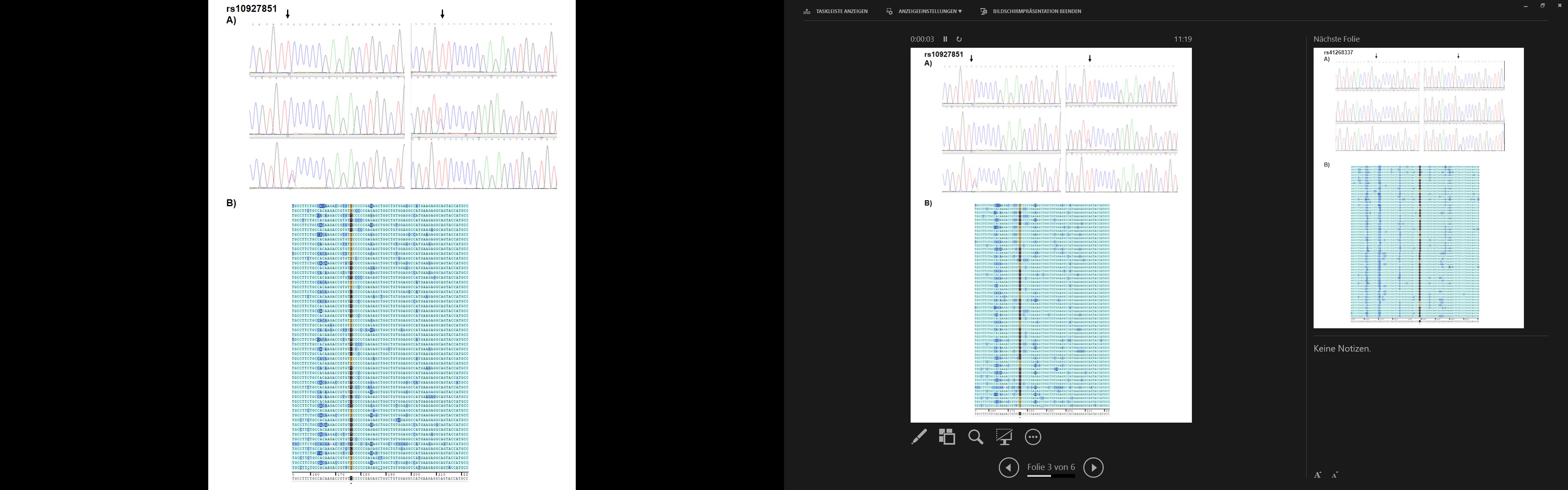


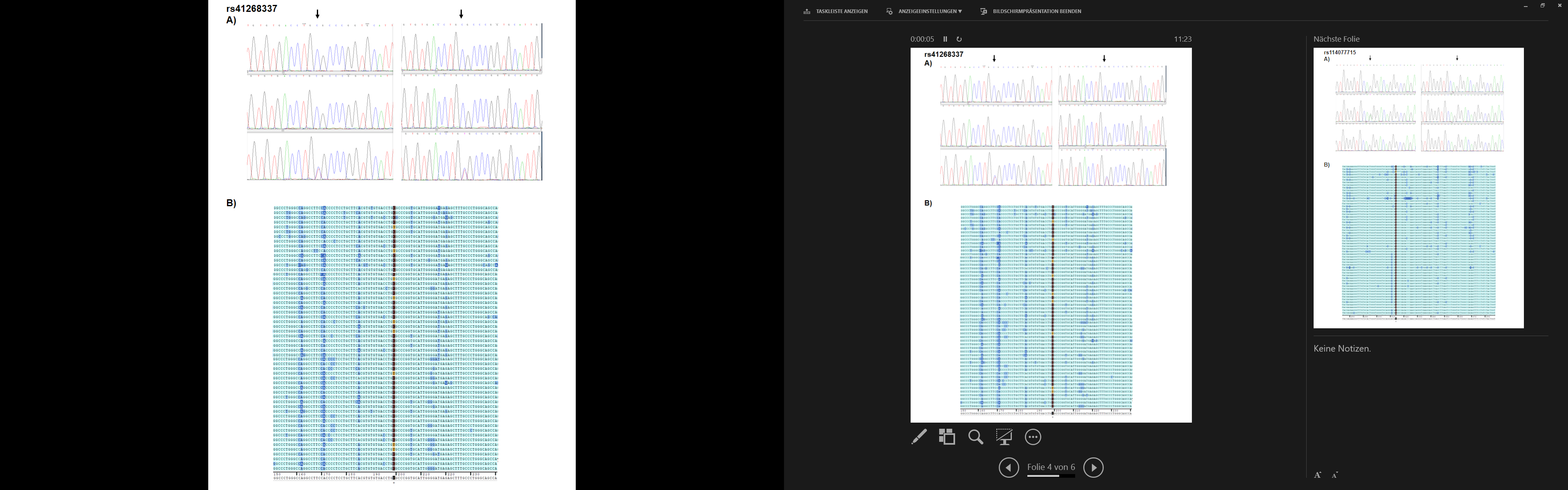


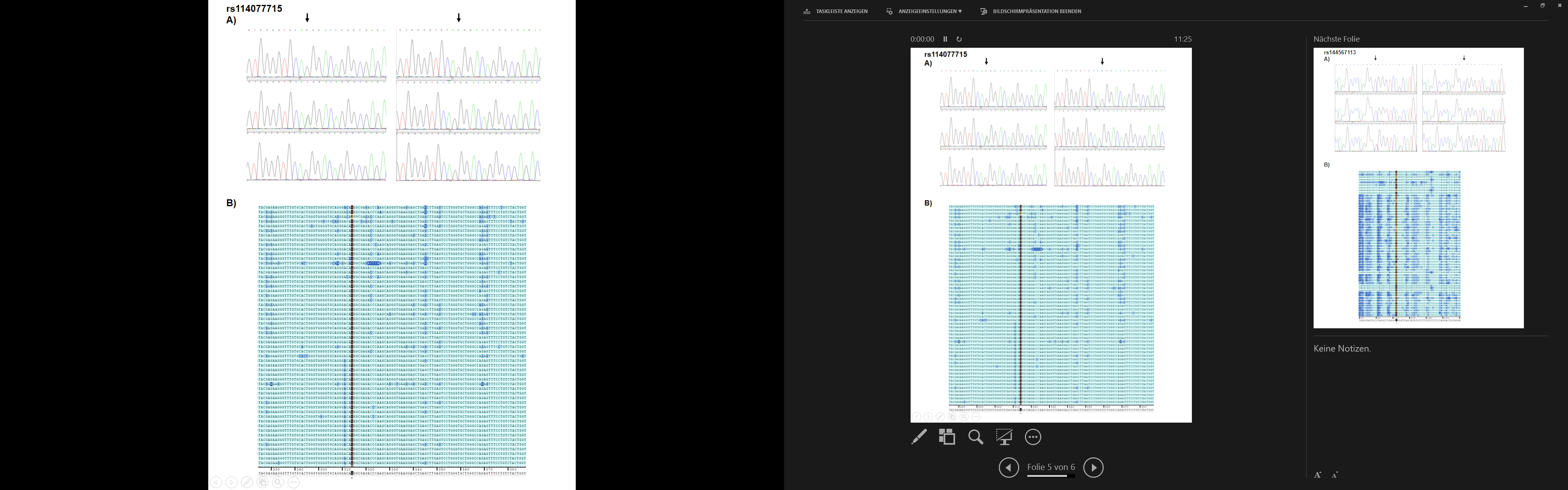


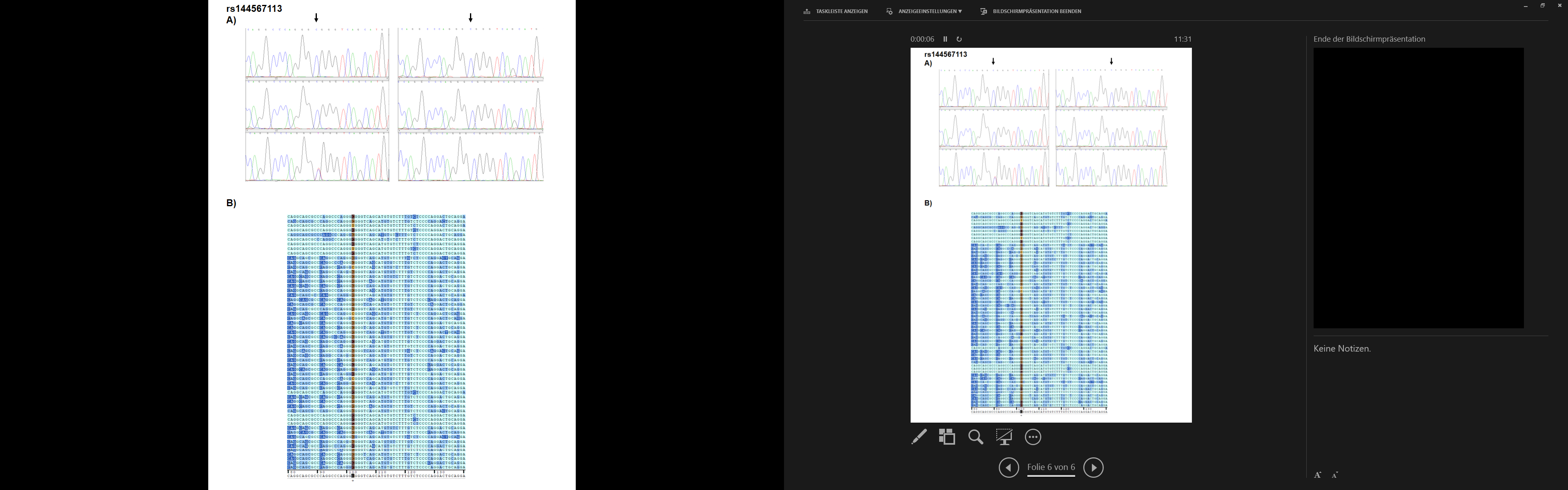

Supplement: Supplementary file 1 — Additional file 1. [file 12881_2020_1037_MOESM1_ESM.docx]
